# Versatile end effector for laparoscopic robotic scrub nurse

**DOI:** 10.1007/s11548-023-02892-4

**Published:** 2023-05-08

**Authors:** Lars Wagner, Sven Kolb, Christian Looschen, Lukas Bernhard, Jonas Fuchtmann, Maximilian Berlet, Johannes Fottner, Alois Knoll, Dirk Wilhelm

**Affiliations:** 1 Research Group MITI, University Hospital rechts der Isar, Technical University of Munich, Munich, Germany; 2 Chair of Materials Handling, Material Flow, Logistics, Technical University of Munich, Munich, Germany; 3 Department of Surgery, University Hospital rechts der Isar, Technical University of Munich, Munich, Germany; 4 Chair of Robotics, Artificial Intelligence and Real-Time Systems, Technical University of Munich, Munich, Germany

**Keywords:** Robotic scrub nurse, Robotic end effector, Laparoscopic instruments, Surgical robotics

## Abstract

**Purpose:**

Integrating robotic scrub nurses in the operating room has the potential to help overcome staff shortages and limited use of available operating capacities in hospitals. Existing approaches of robotic scrub nurses are mainly focused on open surgical procedures, neglecting laparoscopic procedures. Laparoscopic interventions offer great potential for the context-sensitive integration of robotic systems due to possible standardization. However, the first step is to ensure the safe manipulation of laparoscopic instruments.

**Methods:**

A robotic platform with a universal gripper system was designed to pick up and place laparoscopic as well as da Vinci$^{\circledR }$ instruments in an efficient workflow. The robustness of the gripper system was studied using a test protocol, which included a force absorption test to determine the operational safety limits of the design and a grip test to determine the system performance.

**Results:**

The test protocol shows results regarding force and torque absorption capabilities of the end effector, which are essential when transferring an instrument to the surgeon to enable a robust handover. The grip tests show that the laparoscopic instruments can be safely picked up, manipulated and returned independent of unexpected positional deviations. The gripper system also enables the manipulation of da Vinci$^{\circledR }$ instruments, opening the door for robot–robot interaction.

**Conclusion:**

Our evaluation tests have shown that our robotic scrub nurse with the universal gripper system can safely and robustly manipulate laparoscopic and da Vinci$^{\circledR }$ instruments. The system design will continue with the integration of context-sensitive capabilities.

## Introduction

**Table 1:** Existing robotic scrub nurse systems for open and laparoscopic surgery

RSN system	Year	End effector technology	Use case	Sterility concept	Da Vinci$^{\circledR }$ interoperability
Kochan et al. [2]	2005	Electromagnetic gripper	Open surgery	–	–
Takashima et al. [3]	2008	Magazine	Laparoscopic surgery$^{1}$	$\checkmark$	–
Carpintero et al. [4]	2010	Electromagnetic gripper	Open surgery	–	–
Yoshimitsu et al. [5]	2010	Magazine	Laparoscopic surgery$^{2}$	–	–
Jacob et al. [6]	2012	Latex encapsulated magnetic gripper	Open surgery	($\checkmark$)	–
Kogkas et al. [7]	2019	Electromagnetic gripper	Open surgery	–	–
Muralidhar et al. [8]	2021	Form and force closure system	Open surgery	–	–
SASHA-OR	2023	Dual form and force closure system	Laparoscopic surgery	$\checkmark$	$\checkmark$

1.Handles different types of forceps

2.Handles a laparoscope, forceps and other unspecified laparoscopic instruments

In the operating room (OR), surgical assistants are an essential part of the operating team and are therefore subject to a set of advanced requirements regarding qualification, performance and availability. Unfortunately, a shortage of highly qualified personnel in this field [1] limits the use of available operating capacities in hospitals. Therefore, an increasing number of untrained personnel are recruited, whose inexperience has a considerable influence on the workflow of a surgery. To face these problems, both robotic scrub nurses (RSN) [2–8] and robotic circulating nurses (RCN) [9] have been developed in the last two decades, to assume nursing tasks within the sterile as well as the non-sterile area of the OR.

As laparoscopic surgery has become the gold standard for a number of surgical procedures such as cholecystectomies, appendectomies, inguinal hernia repairs, and some types of colon surgery [10], it offers great potential for the use of such robotic scrub nurse systems [11]. As an example, 190 980 cholecystectomies were performed in Germany in 2020 [12], of which around 90% were performed laparoscopically [13].

Therefore, the aim of the Situation Aware Sterile Handling Arm for the Operating Room (SASHA-OR) research project is to design and implement a RSN to perform tasks in the sterile field of the operating room. The emphasis of the RSN is on assisting the laparoscopic surgical workflow where the surgeon is focused on the situs. In order not to interfere with the workflow, the RSN should ensure a seamless transfer of instruments into the surgeon’s hand and anticipate instrument requests without verbal communication, allowing the surgeon to keep his eyes and concentration on the situs. The focus of this work is on the development of an end effector, required for instrument handovers, which is capable of preparing, transferring, and receiving laparoscopic instruments, sterile goods and surgical specimens, as well as preparing teleoperated systems (e.g., da Vinci$^{\circledR }$ Surgical System) for routine laparoscopic procedures. The robot–human instrument handover, anticipation of instruments and interaction with a RCN will be investigated in later work.

So far, there are already approaches for handling instruments for open surgery. These instruments were manipulated by other RSN systems using an electromagnetic gripper, since they are made of stainless steel [6, 7]. For instruments made of plastic, modifications were performed by attaching a magnetizable steel band [14]. Other RSN systems used a two-finger parallel gripper to manipulate the instruments [8]. A different approach was followed by Heibeyn et al. [15] using a robotic form and force closure clamping gripper to automatically perform postoperative reprocessing of instruments for open surgery. When considering laparoscopic interventions, an electromagnetic gripper is not suitable for the manipulation of instruments due to material and geometric properties. Hence, our approach presented in the following expands on the idea of a form and force closure clamping gripper, while specifically focusing on the handling of laparoscopic and da Vinci$^{\circledR }$ instruments and considering the need for sterility.

Table 1 summarizes previous work on RSNs, their end effector technologies, fields of application, sterility considerations, and the potential for collaboration with the da Vinci$^{\circledR }$ system and compares it with our system (SASHA-OR). While two approaches of RSNs for laparoscopic surgery already exist, the presented solutions can only manipulate certain types of laparoscopic instruments. In addition, none of the presented systems offer the capability for robot-robot interaction with the da Vinci$^{\circledR }$ Surgical System, which would open the door for single surgeon surgeries. In order to fill this knowledge gap, this paper presents our results of the development of our robotic platform and the custom-designed sterile end effector for manipulating both laparoscopic and da Vinci$^{\circledR }$ instruments using a dual form and force closure clamping system. Following various tests in which we compare the capabilities of our system to a benchmark end effector, conclusions are drawn regarding the design of end effectors for use in hygiene-safety critical environments.

## Methods

### Robotic platform

**Fig. 1:**
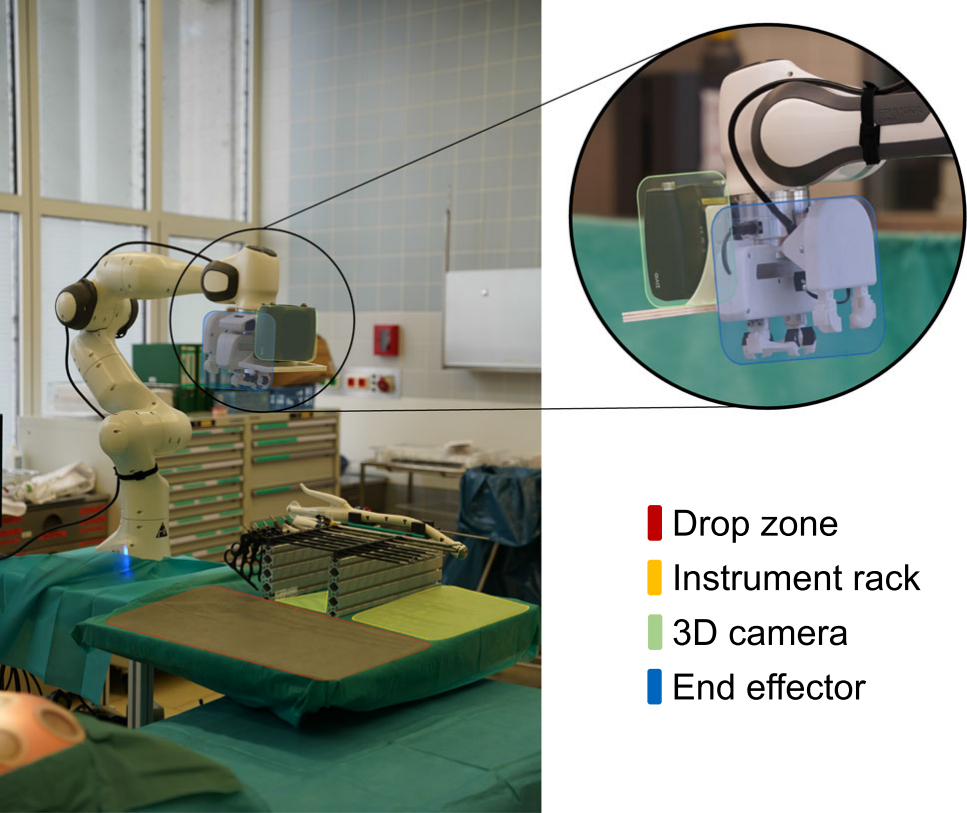
SASHA-OR system consisting of drop zone (red), instrument rack (yellow), 3D camera (green), end effector (blue)

For the handover of instruments to the surgeon, we designed a platform where a robotic handling arm (Panda, *Franka Emika GmbH*, Germany) is positioned on a mobile base with a surgical tray attached. The tray contains a drop zone (DZ) and an instrument rack (IR), in which the laparoscopic instruments are stored at predefined positions. The bearing of the instruments at the beginning and end of the instrument shaft ensures a simple robotic pick-up and return of the instrument. The handling arm is placed behind the surgical tray so that its range of motion covers the entire tray. The working areas of the surgeon and the handling arm overlap, so that instruments can be transferred. The mobile base provides maneuverability, while foot stamps allow the robotic platform to be securely fixed next to the operating table. The end effector and 3D camera (Zivid Two, *Zivid*, Norway) are mounted to the robotic arm for manipulation and recognition of the laparoscopic instruments. Using speech recognition, the surgeons are able to communicate verbally with the robotic platform. Figure 1 shows the prototypical robotic platform with the instrument rack, the drop zone, the end effector mounted to the Franka Emika Panda handling arm, as well as a detailed view of the end effector.

### Workflow

**Fig. 2:**
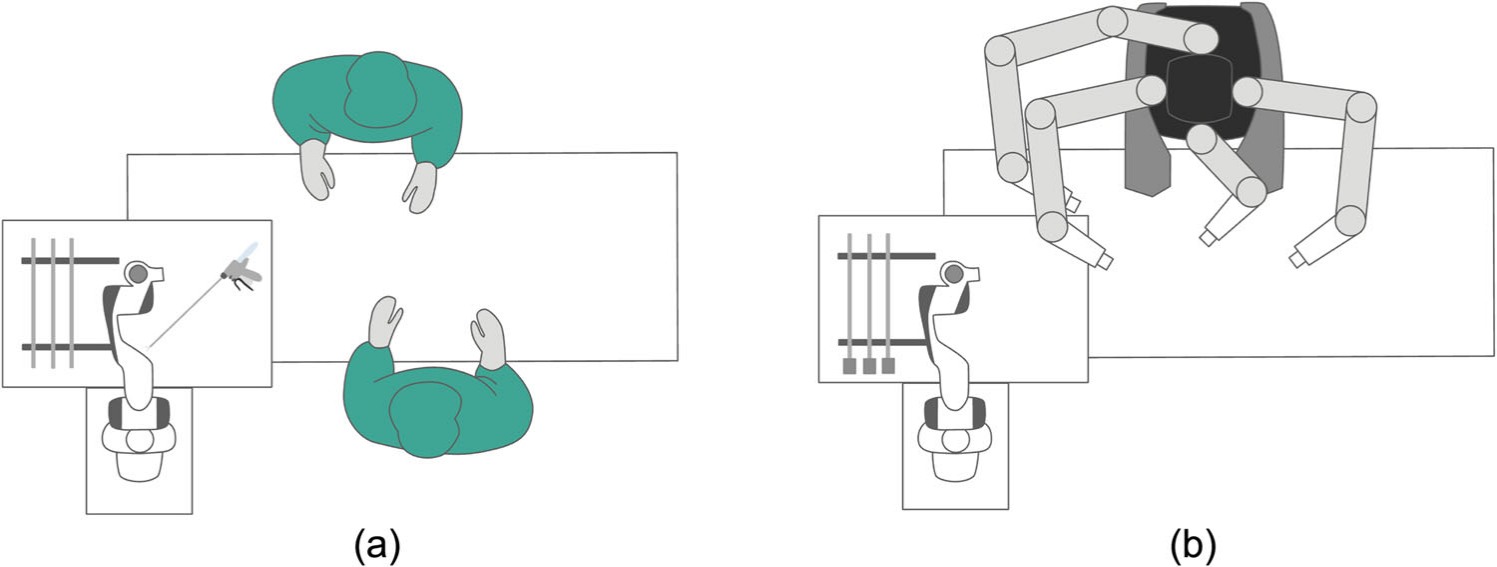
Schematic setups of sterile operating areas for interventions with the SASHA-OR system, **a** laparoscopic surgery, **b** robotic surgery

Since the robotic platform has one arm, the instrument handover process needs to be carefully planned to ensure fast and efficient instrument transfer. For this purpose, the return process uses the drop zone on the instrument tray as an intermediate storage area to enable a faster handover of the required instrument. In the following we briefly describe the handover strategy, as it is relevant for the methodology of the grip tests. The SASHA-OR setup is schematically shown in Fig. 2 for laparoscopic and robotic interventions.

#### Instrument handover

An instrument is either requested by the surgeon via voice or gesture command, or the robotic assistant decides to initiate the handover process. Using tactile sensors and computer vision methods, the system is supposed to act in a context-sensitive manner allowing the transfer process to be initiated autonomously. The instrument is located in the instrument rack and is picked up by the robotic assistant at the predefined contact point. Using dynamic path planning, the robotic assistant moves the instrument to the predefined handover point while visually monitoring the transfer space to prevent collisions. To receive the instrument, the surgeon applies a manual force on the end effector, e.g., by pulling the instrument handle. When the force exceeds a predefined value, the robotic assistant releases its grip. The handover process is complete when the instrument is received by the surgeon.

#### Pick-up of used instruments

The surgeon places a used instrument within the predefined drop zone. The robotic assistant recognizes and locates the instrument via the 3D camera. Using the end effector, the robot picks up the used instrument or sterile item at the predefined contact point. Using dynamic path planning, the robotic assistant moves the used instrument back to the instrument rack while visually monitoring the transfer space to prevent collisions. The drop zone is designed to speed up the exchange process, as the robotic arm can provide a required instrument in advance, rather than having to receive the exchanged instrument first.

#### Interaction with da Vinci$^{\circledR }$ surgical system

During interaction with the da Vinci$^{\circledR }$ System, no surgeon is needed. The robot picks up the da Vinci$^{\circledR }$ instrument from the instrument rack by gripping the shaft of the instrument, stabilizes the instrument head and inserts the instrument into one of the da Vinci$^{\circledR }$ arms. During this process, the instrument’s release buttons are pressed to ensure locking of the instrument into the arm. When the instrument is removed from the da Vinci$^{\circledR }$ arm, the release buttons are pressed again, the instrument is pulled out of the arm and placed back on the instrument rack. The instrument head needs to be stabilized both during the insertion and removal process.

**Fig. 3:**
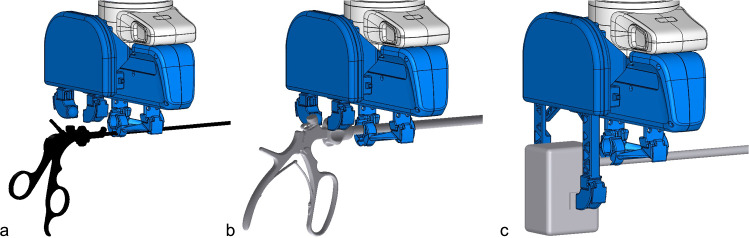
End effector (blue) consisting of two subsystems (shaft gripping subsystem and release button subsystem); **a** shaft gripping subsystem manipulating instruments with lower gripping area (LGA), **b** shaft gripping subsystem manipulating instruments with upper gripping area (UGA), **c** shaft gripping subsystem manipulating da Vinci$^{\circledR }$ instrument with lower gripping area (LGA) and release button subsystem stabilizing da Vinci$^{\circledR }$ instrument head while pressing the release buttons

### End effector

For the manipulation of laparoscopic instruments, we designed a universal electrically actuated clamping end effector with separate gripping areas for adaptation to instruments of different shaft diameters. The end effector is capable of securely grasping the cylindrical shafts of laparoscopic and da Vinci$^{\circledR }$ instruments via force and form closure. The standardization of laparoscopic instrument diameters and simple cylindrical geometry make the instrument shaft an ideal target for robotic manipulation. Another advantage of targeting the shaft is the ability to grasp the instruments near their center of gravity, minimizing the occurrence of tilting moments and other undesirable forces during manipulation [16].

The shaft gripping subsystem has flexible grip covers on the functional surfaces of the upper gripping area (UGA) to prevent larger instruments, such as the stapler, from slipping off. The lower gripping area (LGA) is used to grip laparoscopic instruments with an instrument shaft diameter of 5–10 mm, while the UGA is used to manipulate instruments with an instrument shaft diameter of 10–20 mm.

The functional surfaces of the LGA are purposefully left uncovered, as the axial rotation of the 5 mm instruments such as scissors or forceps, resulting from the low-friction material pairing and the uncentered mass of the instrument handles, acts as a passive gravity-assisted payload orientation system, ensuring a homogenous orientation of instruments after pickup from the drop zone. This means that the instrument handle automatically aligns downwards allowing a straightforward return of the instrument to the instrument rack. Each gripping area is equipped with two contact points spaced 50 mm apart along the instrument shaft to counteract the occurring tilting moments and to improve the stability and balance of the system. The prototypical end effector uses the existing linear actuation mechanism of the Franka Emika Panda Hand^1^ to actuate the shaft gripping subsystem.

The release button subsystem of the end effector provides the capability to manipulate da Vinci$^{\circledR }$ instruments. In addition to an increased payload stability through the fixation of the freely rotating instrument head, the subsystem enables the autonomous intraoperative mounting and deployment of da Vinci$^{\circledR }$ instruments to the da Vinci$^{\circledR }$ Surgical System through the manipulation of its release buttons, located on the sides of the instrument head. The vertical range of motion ensures a compact storage configuration of the release button subsystem when not in use.

**Fig. 4:**
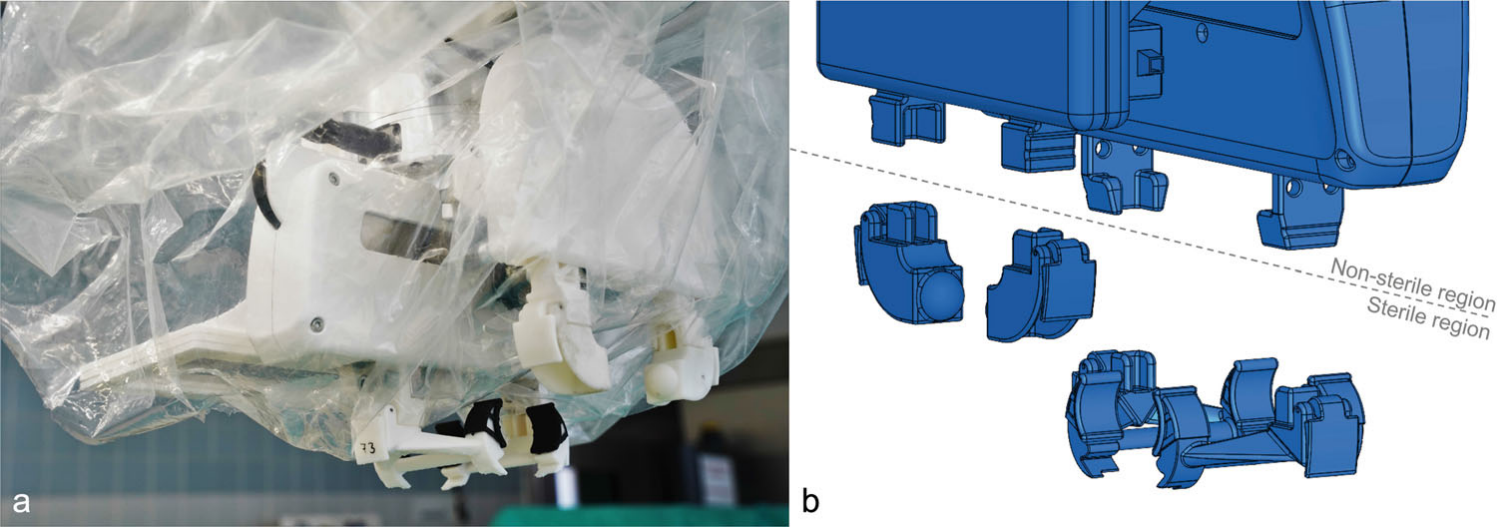
**a** End effector under sterile conditions, **b** Exploded view of the end effector indicating the position of the sterile cover, thus dividing the end effector into a sterile and a non-sterile region

To ensure conformity with the sterility guidelines present in a hospital setting, a sterility concept was developed for the end effector. The concept provides a two-part design with a sterile and a non-sterile region, separated by a sterile cover. The sterile cover is inserted between the top and bottom joint via a hinged clasping mechanism. The sterile end effector is attached to the mounting flange of the handling arm via a custom mount, designed for use together with the corresponding custom mount for the 3D camera system, which is attached on the opposite side of the flange. Figure 3 shows the end effector gripping various instruments, while Fig. 4 illustrates the sterility concept described above, where only the final parts of the end effector were designed as sterile single-use components.

All components of the prototypical gripper system were manufactured via Fused Deposition Modeling (FDM), apart from the final parts of the release button subsystem and parts of the clasping mechanism, which were printed via stereolithography (SLA) due to their small dimensions and high accuracy of functional surfaces [17].

### Load absorption tests

**Fig. 5:**
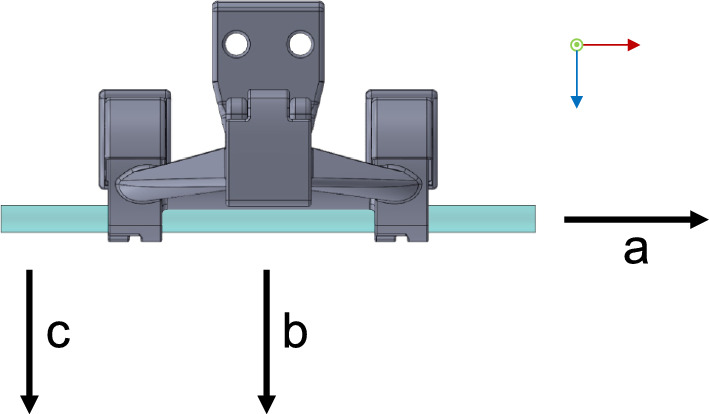
Force application modeling for payload translation and rotation (black arrows indicate applied force direction), **a** force absorption in x direction, **b** force absorption in z direction, **c** Torque absorption in the xz plane

When transferring an instrument based on the workflow in “Workflow” section, manual forces are applied to the end effector by the surgeon to signal the system that it can release the instrument. These must be resisted by the shaft gripping subsystem for safety reasons. For a dropped instrument, a new sieve would have to be opened due to hygiene regulations, causing additional costs. Therefore, the objective of the load absorption tests is to evaluate the operational safety limits of the design. For this purpose, we defined various test scenarios that apply individual forces and torques to the shaft gripping subsystem that may occur during instrument handover. These include force absorption capabilities in x- and z-direction and torque absorption capabilities in the xz plane. Figure 5 illustrates the modeling of the load absorption tests. The black arrows indicate the applied force direction of each test.

All tests were performed with dummy instruments with shaft diameters of 5 mm (LGA) and 20 mm (UGA). Twenty test runs were performed for each instrument. The applied forces were measured using a Sauter FH 500 and recorded via MATLAB.^2^ During the test scenarios, the instrument dummys were inserted between the prototypical end effector fingers at the appropriate gripping area, which applied a gripping force of 70 N corresponding to the maximum continuous grasping force [18] of the Franka Emika Panda Hand. The force measuring device was slowly pulled or pushed in the defined direction until the dummys were dislodged from the grip of the prototypical end effector.

**Fig. 6:**
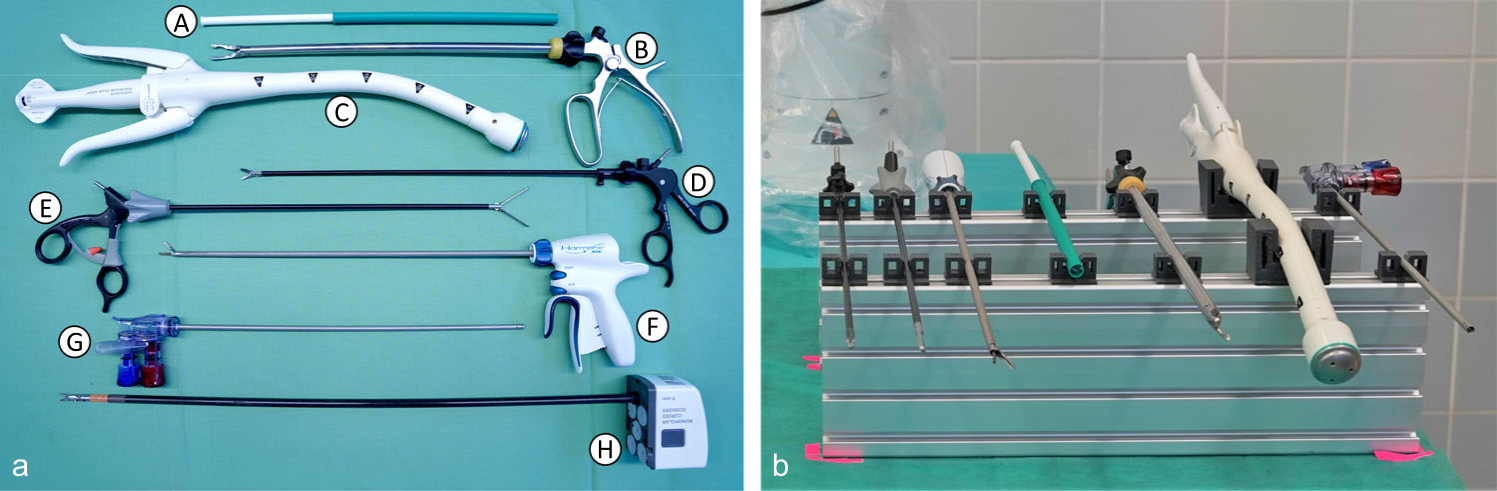
**a** Laparoscopic instruments (ID: A - G) and da Vinci$^{\circledR }$ instrument (ID: H), **b** laparoscopic instruments stored in a prototypical instrument rack with flexible brackets

**Table 2:** Instrument allocation

Gripping area	Instrument	Manufacturer	Mass	ID
Upper gripping area	Retrieval pouch	Homeport	13.2g	A
	Clip applicator	Ethicon	268.4g	B
	Stapler	Covidien	475.4g	C
Lower gripping area	Metzenbaum scissors	Karl Storz	77.9g	D
	Bowel grasping forceps	Aesculap	98.8g	E
	Ultrasonic dissector	Ethicon	133.6g	F
	Suction and irrigation device	Applied Medical	53.0g	G
Lower gripping area, release button subsystem	da Vinci$^{\circledR }$ instrument	Intuitive Surgical	187.5g	H

**Table 3:** Task success criteria

Category	Pick$^{1}$	Place$^{2}$
Success	Instrument gripped securely in the expected orientation	Instrument inserted correctly into the appropriate slot
Partial success	Instrument gripped in an unexpected orientation, but still secure	Instrument partially inserted into the correct slot
Failure	Instrument not gripped or instrument dropped	Instrument not inserted securely or collision with another instrument or object

1. Pick refers to the process of grasping an instrument from the instrument rack or the drop zone

2. Place refers to the process of placing an instrument back into the instrument rack

### Grip tests

#### Pick-and-place test

To evaluate the main task of the robotic handling arm during operation, we defined grip tests to verify the system performance in an application context. These include a benchmarked verification of the principal handling capability of the prototypical end effector as well as an examination of the robustness against unexpected positional deviation of the payload. The aim of the pick-and-place test is to compare the performance of the prototypical end effector against the standard Franka Emika Panda gripper in an application-specific scenario. The test examines the grasping capabilities of both end effectors from the instrument rack and the drop zone as well as the placing of instruments into the instrument rack. The test is divided into two steps, in which first the handling capabilities of the laparoscopic instruments and then those of the da Vinci$^{\circledR }$ instruments were examined.

For this purpose, we stored various laparoscopic instruments commonly used in cholecystectomies and sigmoid resections in a prototypical instrument rack with flexible brackets, which is illustrated in Fig. 6. The flexible brackets lock the instruments in place but allow for easy removal of the instruments by the robot (max. removal force $5.8 {N} \pm 0.7 {N}$). The spacing between the different instrument slots is optimized for compact fitting of all necessary instruments, which are listed in Table 2. In the first step, the laparoscopic instruments were picked from the instrument rack, manipulated and subsequently returned to their allocated slots in the instrument rack. Subsequently, the picking capabilities of the end effector from the drop zone were evaluated. For this purpose, the laparoscopic instruments from Table 2 were placed in a defined position in the drop zone and picked by the robot. For every instrument, each task was performed 10 times using the prototypical end effector and 10 times using the standard Franka Emika Panda gripper as a benchmark. The success of a task is defined in three categories based on a selection of relevant criteria, shown in Table 3.

**Table 4:** Failure conditions

Condition	Reason
Success rate (*SR*) $< 90 \%$	No more than one partially successful grip is acceptable (only if manipulation is still possible)
Failure rate $> 0 \%$	A failed grip is unacceptable due to safety considerations
Geometric limit reached (end of instrument shaft or maximum opening width)	The effector at the instrument tip is delicate and should not be grasped; the maximum gripper opening width presents a physical limitation

In the second step, the handling capability of da Vinci$^{\circledR }$ instruments was evaluated by running through the same routine as in the previous step, with additional deployment of the release button subsystem as illustrated in Fig. 3. For interaction with the da Vinci$^{\circledR }$ robot, actuation of the release buttons and stabilization of the instrument head must be possible. Since actuation of the buttons of the da Vinci$^{\circledR }$ instrument and stabilization of the instrument head is only possible using our end effector, the performance of the benchmark gripper when manipulating a da Vinci$^{\circledR }$ instrument was not evaluated due to insufficient functional fulfillment.

#### Robustness against positional deviation

As described in “Pick-up of used instruments” section on the workflow for returning an instrument, the handling arm recognizes the instrument via a 3D camera. The robot should then pick up the instrument and return it to the instrument rack. To assess potential tolerances in the recognition and pickup process, we evaluate the robustness against unexpected positional deviation in this scenario. A quantification of the maximum allowable deviation is relevant in order to define the necessary picking precision of the system. As the instrument rack presents a defined positioning system for the contained instruments, it is sufficient to evaluate picking performance from the drop zone.

**Fig. 7:**
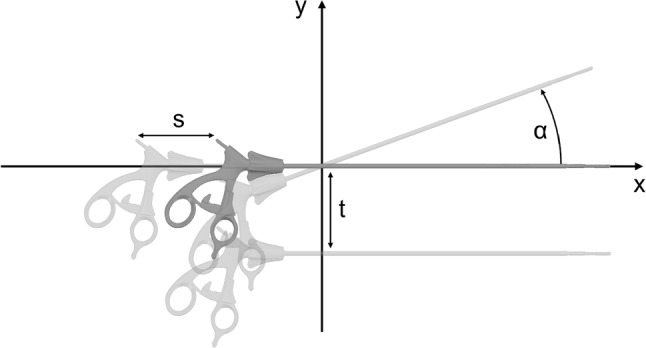
Depiction of axial translation *s*, transversal translation *t*, and transversal rotation $\alpha$

The experiment is divided into three subtests, covering axial translation *s*, transversal translation *t* as well as transversal rotation $\alpha$. The respective displacement directions are indicated in Fig. 7.

The evaluation is performed by grasping the forceps (E) with the LGA and the clip applicator (B) with the UGA. In addition, the stapler (C) is evaluated due to its position as largest, heaviest, and therefore most critical instrument. An iterative evaluation of the gripping positions is conducted in 5mm or 5$^{\circ }$ steps, depending on the test type. For each position, 10 test runs are performed. This evaluation is continued until one or more of the failure conditions listed in Table 4 is fulfilled. In that case, the previous increment is considered as the deviation limit.

## Results

### Load absorption tests

**Fig. 8:**
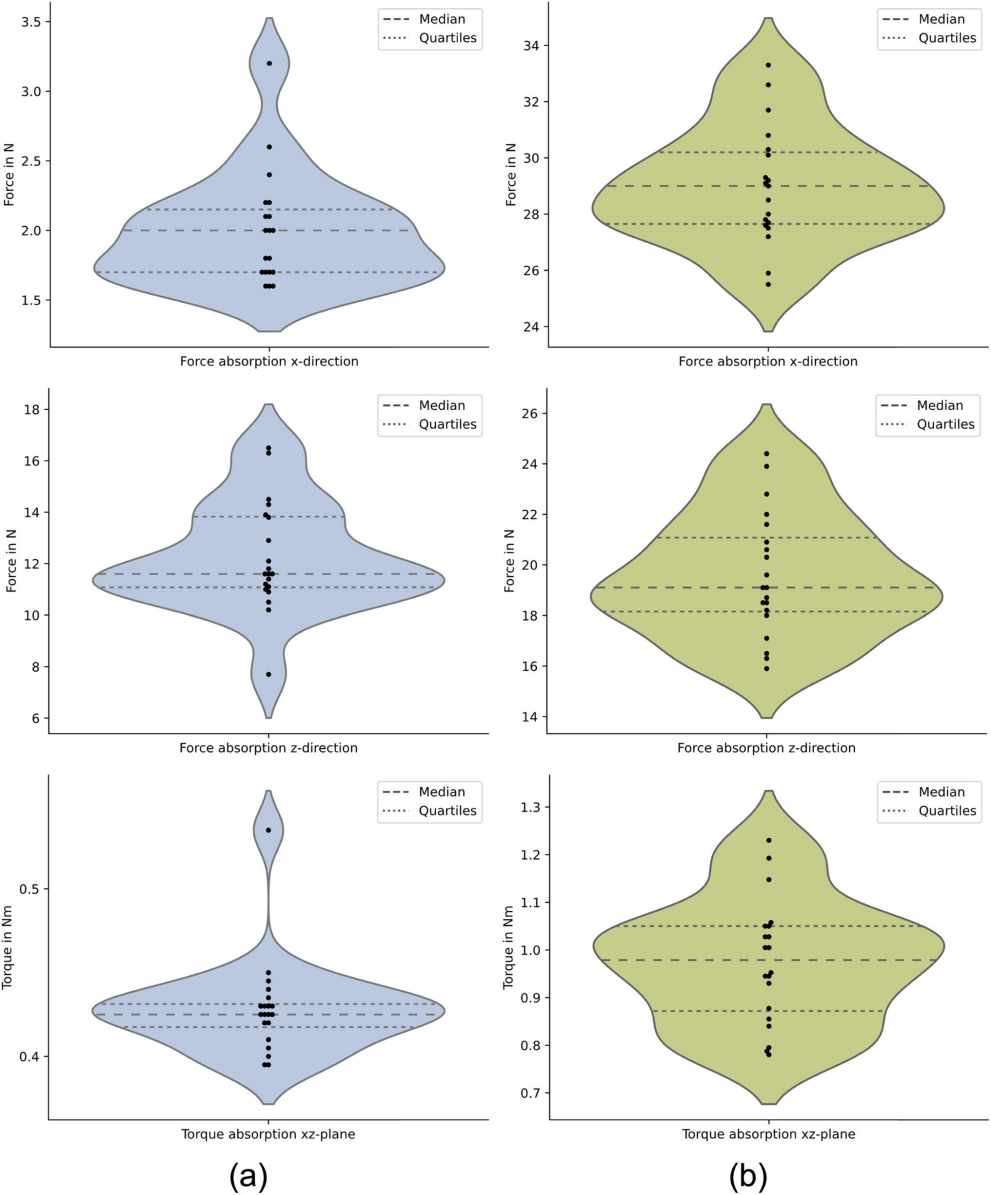
**a** Load absorption tests for lower gripping area (LGA), **b** Load absorption tests for upper gripping area (UGA)

As described above, force and torque tests were performed to evaluate the operational safety limits. The results of the load absorption tests are shown in Fig. 8. The results of the tests in the x-direction demonstrate the effect of the flexible grip covers of the UGA, providing high adhesion of the instrument in the prototypical end effector. The median force absorption at the UGA reaches $29.0 \textrm{N} \pm 4.14 \textrm{N}$, while the median force absorption at the LGA reaches $2.0 \textrm{N} \pm 0.16 \textrm{N}$, leading to a median ratio of $k_{P,x} = 14.5$.

The quantitative data obtained in the tests in z-direction proves that the force absorption capability of the shaft gripping subsystem in z-direction is dependent on the gripping area, as the median force absorption at the UGA ($19.10 \textrm{N} \pm 2.38 \textrm{N}$) is around $k_{P,z} = 1.65$ times higher than the median value at the LGA ($11.60 \textrm{N} \pm 2.06 \textrm{N}$). The dispersion of the data can be considered moderate.

Furthermore, the torque absorption capability in the xz plane is strongly dependent on the gripping area, as the median torque absorption at the UGA ($0.98 \textrm{Nm} \pm 0.12 \textrm{Nm}$) is around $k_{P,xz} = 2.30$ times higher than the median value at the LGA ($0.43 \textrm{Nm} \pm 0.03 \textrm{Nm}$). The results indicate a very low dispersion of torque absorption at the LGA. At the UGA, the dispersion of the measured data is noticeably higher.

### Grip tests

To ensure adequate system performance in an application context, we performed an evaluation of the pick-and-place capabilities of the end effector, as well as an examination of the system robustness against positional deviations, both translational and rotational. The results of the pick-and-place test are shown in Table 5.

To improve the visual representation of relevant information, only the instrument groups for which the results of the two grippers were different are depicted. All other groups yielded identical results with $SR = 100 {\%}$. The results show that the benchmark gripper had severe issues handling the stapler (C), especially during pick tasks, whereas the prototypical end effector was able to manipulate this instrument with no problems. The ultrasonic dissector (F) also posed problems for the benchmark gripper during the place routine due to the relatively tight instrument slot tolerances, as well as during the transfer zone pick routine. The prototypical end effector handled this instrument with no issues.

Furthermore, we were able to verify the extended da Vinci$^{\circledR }$ instrument (H) manipulation capability of the prototypical end effector using the release button subsystem, as the success rates of the pick-and-place test demonstrate ($SR_{P,IR,pick,dV} = SR_{P,IR,place,dV} = SR_{P,DZ,pick,dV} = 100 {\%}$). Through the release button system, the end effector was able to actuate the buttons of the da Vinci$^{\circledR }$ instruments as it was manipulated, thus stabilizing the instrument head. As described in “Pick-and-place test” section, the benchmark gripper was not evaluated due to insufficient functional fulfillment regarding da Vinci$^{\circledR }$ instrument manipulation.

**Table 5:** Pick-and-place test results (only instrument groups with different behaviors shown

Routine	ID	Gripper	Success	Partial success	Failure
Pick from instrument rack	C	P	$100 {\%}$	$0 {\%}$	$0 {\%}$
		B	$0{\%}$	$100 {\%}$	$0 {\%}$
	HD$^1$	P	$100 {\%}$	$0 {\%}$	$0 {\%}$
Place in instrument rack	F	P	$100 {\%}$	$0 {\%}$	$0 {\%}$
		B	$0 {\%}$	$100 {\%}$	$0 {\%}$
	G	P	$100 {\%}$	$0 {\%}$	$0 {\%}$
		B	$0 {\%}$	$100 {\%}$	$0 {\%}$
	H$^1$	P	$100 {\%}$	$0 {\%}$	$0 {\%}$
Pick from drop zone	F	P	$100 {\%}$	$0 {\%}$	$0 {\%}$
		B	$80 {\%}$	$20 {\%}$	$0 {\%}$
	C	P	$100 {\%}$	$0 {\%}$	$0 {\%}$
		B	$0 {\%}$	$100 {\%}$	$0 {\%}$
	H$^1$	P	$100 {\%}$	$0 {\%}$	$0 {\%}$

1.Performance of benchmark gripper when manipulating da Vinci$^{\circledR }$ instruments was not evaluated due to insufficient functional fulfillment

**Fig. 9:**
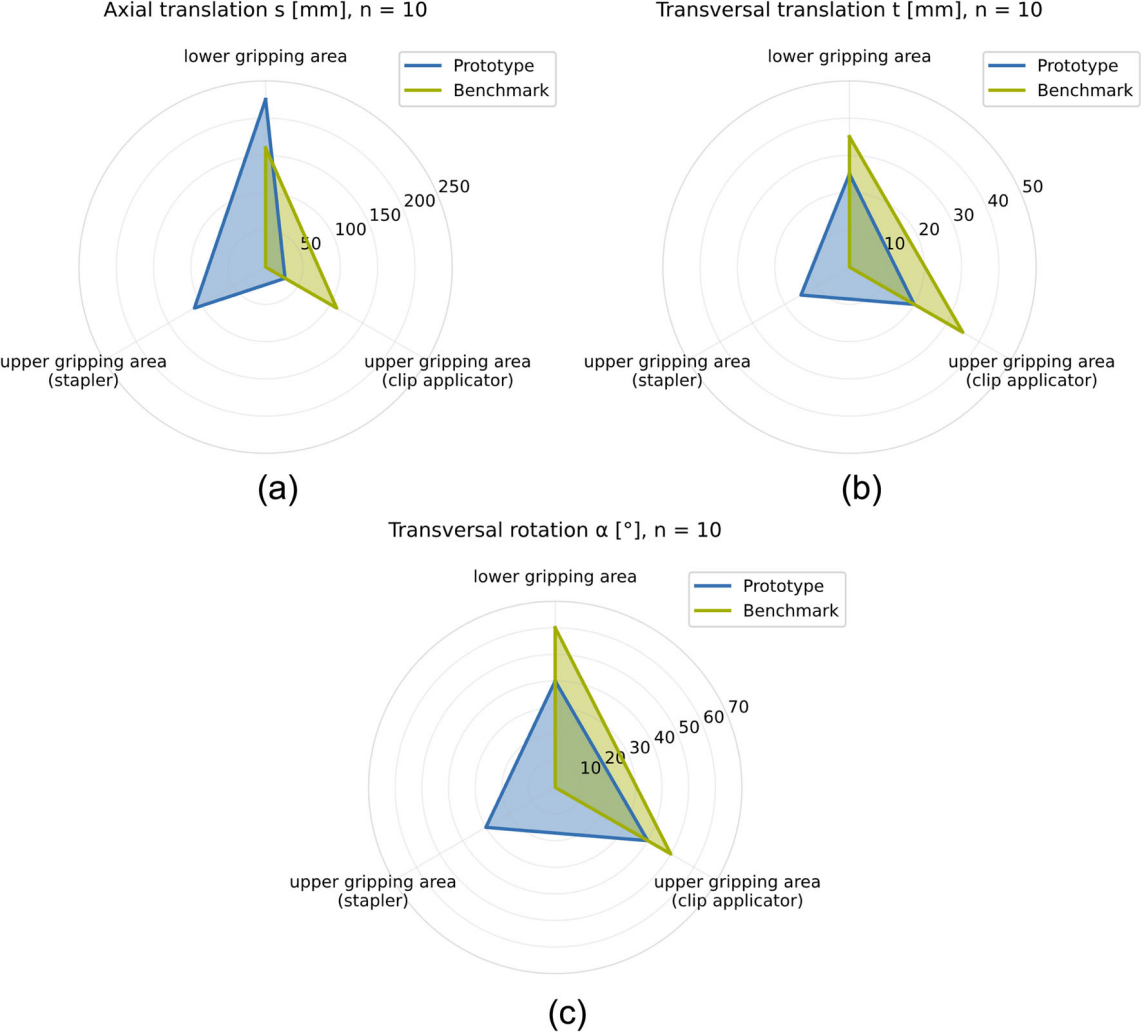
**a** Robustness against axial translation *s*, **b** Robustness against transversal translation *t*, **c** Robustness against transversal rotation $\alpha$

In the second step, we examined the robustness against unexpected positional deviation of the payload. The results, shown in Fig. 9, indicate that the robustness against axial translation along the shaft is generally considerably higher for the prototypical end effector ($s_{P,LGA} = 225 {\mathrm{\,mm}}$, $s_{P,UGA,C} = 110 {\mathrm{\,mm}}$) than for the benchmark gripper ($s_{B,LGA} = 160 {\mathrm{\,mm}}$, $s_{B,UGA,C} = 0 {\mathrm{\,mm}}$), except when picking the clip applicator ($s_{P,UGA,B} = 30{\mathrm{\,mm}}$, $s_{B,UGA,B} = 110 {\mathrm{\,mm}}$). This behavior is caused by the geometry of the end effector, which prevents it from sliding underneath the shaft of the clip applicator further down the shaft. The robustness against transversal translation is generally high for both end effectors, although the absolute limits of the benchmark gripper ($t_{B,LGA} = t_{B,UGA,B} = 35 {\mathrm{\,mm}}$) are slightly higher due to geometric constraints compared to the prototypical end effector ($t_{P,LGA} = 25{\mathrm{\,mm}}$, $t_{P,UGA,B} = 20 {\mathrm{\,mm}}$). A similar result is obtained for the robustness against transversal rotation, which is generally high for both end effectors, although the absolute angular limits of the benchmark gripper ($\alpha _{B,LGA} = 60^{\circ }$, $\alpha _{B,UGA,B} = 50^{\circ }$) are again slightly higher due to geometric constraints ($\alpha _{P,LGA} = 40^{\circ }$, $\alpha _{P,UGA,B} = 40^{\circ }$). The two separate contact points of the prototype greatly improve grip stability, but take up more space around the instruments, thus limiting the maximum rotation angle. The robustness evaluation provides evidence that there is no preferable orientation in which it is possible for the benchmark gripper to handle the stapler successfully ($t_{P,UGA,C} = 15 {\mathrm{\,mm}}$, $t_{B,UGA,C} = 0 {\mathrm{\,mm}}$, $\alpha _{P,LGA,C} = 30^{\circ }$, $\alpha _{B,LGA,C} = 0^{\circ }$) in contrast to the prototype, which shows a high robustness to axial and transversal translation as well as transversal rotation of the stapler.

## Discussion

### Subsystem performance and safety aspects

In the load absorption tests, we evaluated the performance of the shaft gripping subsystem under the application-specific load scenarios defined in Fig. 5. The results show that the force and torque absorption capabilities of the end effector are sufficient for safe and robust operation. The consistently higher values achieved when loading the UGA concur with the planned gripping approach, as this gripping area targets instruments with a larger diameter, which generally have a larger mass and therefore require a higher force resistance than the smaller diameter instruments. Nevertheless, further evaluations must verify whether these absorption capabilities are sufficient for the handover of instruments to the surgeon.

### System performance and robustness

In the grip tests, we evaluated the performance of the system in an application context. The results show that the prototypical end effector offers several advantages in comparison with the benchmark gripper. A key benefit is the remarkably better performance when handling the large, heavy stapler (C). While the benchmark gripper was unable to safely pick this instrument ($SR_{B,IR,pick,C}= SR_{B,DZ,pick,C} = 0 {\%}$), the prototypical end effector had no issues with it ($SR_{P,IR,pick,C} = SR_{P,DZ,pick,C} = 100 {\%}$). With all instruments, the prototypical system executed the place task better than the benchmark gripper ($SR_{P,IR,place} = 100 {\%}$, $SR_{B,IR,place} = 0 {\%}$). This can largely be attributed to the two separate contact points of the main end effector spaced 50 mm apart along the instrument shaft, which greatly improve the vertical force application necessary for proper insertion of the instrument into the instrument rack without inducing a tilting moment, as well as considerably increasing the general payload stability.

The observation of the grip test yielded several further advantageous factors of the system, which are not directly represented in the data shown in “Grip tests” section. The main factor is the capability to passively align the instrument handles vertically, which is necessary in order to properly replace instruments from the drop zone into the instrument rack due to the limited space available. The non-covered LGA of the prototype enables a picked instrument to rotate around its axis due to the off-center mass of the instrument handle. The end effector tips of the benchmark gripper, however, prevent the axial rotation of picked instruments.

A further advantage of our prototypical end effector is the increased stability and extended manipulation capability of da Vinci$^{\circledR }$ instruments, the latter of which is attributed to the release button subsystem. The benchmark gripper lacks this feature and thus is unable to manipulate the release buttons of a da Vinci$^{\circledR }$ instrument while simultaneously holding it. As the actuation of the release buttons is necessary for the mounting and deployment of instruments to the da Vinci$^{\circledR }$ Surgical System, our developed end effector incorporates a key requirement for future integration of this functionality.

The results show that our design offers a notably higher robustness against axial translation when handling most instruments except for the clip applicator and slightly lower robustness for transversal translation except for the stapler. We were able to show that the robustness against transversal translation and transversal rotation is generally high for both end effectors, while the geometric constraints given by the maximum opening width of the grippers and their respective geometries were identified as the limiting factor for rotational and translational robustness. In this context, the tolerance the mechanical system can compensate should be sufficient to allow for some degree of uncertainty during computer vision-based recognition of the instruments using a 3D camera. The robustness evaluation further proves the handling of the stapler to be a key advantage of the prototypical end effector over a typical clamping gripper.

## Limitations

One main limitation we identified is the geometry of the shaft gripping subsystem. The results of the grip tests show that the robustness of picking the clip applicator from the drop zone is limited by the fact that the end effector must push underneath the instrument shaft in order to grasp the instrument with the UGA. The robustness of this task could be increased by refining the geometry around the bottom of the end effector to improve the ability to slide underneath the instrument shafts. The end effector geometry is also the limiting factor for the robustness against transversal rotation, and a compact redesign of the end effector could improve the results in this area. One additional factor to benefit from a compact redesign is the pick task from the instrument rack, as relatively large buffer zones are currently required between the instruments (see Fig. 6). A more compact arrangement could free up space for additional instruments on the rack, thus potentially improving the workflow.

Another limitation of the system is the high positional precision required for the release button subsystem to function as intended. As the correction of axial positioning errors of the da Vinci$^{\circledR }$ instruments is limited to the width of the release button due to the operating principle of the system, the positional requirements are higher than for all other instruments. This should not pose a problem for the intended computer vision-based positioning system, yet this factor should be considered during further development and optimization processes.

As the performed tests were focused on evaluating separate load cases, a superposition of these cases was not tested and should be subject of further research for closer approximation of real-world load scenarios such as the robotic handover of an instrument to the surgeon.

In general, a single robotic handling arm limits the efficient transfer of instruments. Compared to a human OR assistant who can work with both hands to pick up and drop instruments at the same time, the return process of our robotic system requires an intermediate storage in order to maintain a fast workflow. This poses challenges for the design of the end effector, as it must be able to pick up the instruments from the drop zone.

## Conclusion

In this paper, we presented our robotic scrub nurse for laparoscopic interventions with a comprehensive evaluation of our universal prototypical gripper system. We conclude that our prototypical end effector ensures safe robotic pick-up and return of laparoscopic instruments from and to the instrument rack. Using a dual form and force closure mechanism with a dedicated release button subsystem, we provide the ability to pick up and actuate the release buttons of da Vinci$^{\circledR }$ instruments to enable robot-robot interaction which opens the door for single surgeon surgeries. In addition, unexpected positional deviations, both translational and rotational, can be compensated by the prototypical end effector when a laparoscopic instrument is picked up from the drop zone. While we identified some limitations offering further potential for improvement, we consider our results to support the general feasibility of robotic scrub nurses for laparoscopic interventions.

## Footnotes

1 Franka Emika Panda Hand (see https://www.franka.de/applications).

2 MATLAB (see https://www.mathworks.com/products/matlab).
