# Correction to: Versatile end effector for laparoscopic robotic scrub nurse

**DOI:** 10.1007/s11548-023-02969-0

**Published:** 2023-06-10

**Authors:** Lars Wagner, Sven Kolb, Christian Looschen, Lukas Bernhard, Jonas Fuchtmann, Maximilian Berlet, Johannes Fottner, Alois Knoll, Dirk Wilhelm

**Affiliations:** 1 Research Group MITI, University Hospital rechts der Isar, Technical University of Munich, Munich, Germany; 2 Chair of Materials Handling, Material Flow, Logistics, Technical University of Munich, Munich, Germany; 3 Department of Surgery, University Hospital rechts der Isar, Technical University of Munich, Munich, Germany; 4 Chair of Robotics, Artificial Intelligence and Real-Time Systems, Technical University of Munich, Munich, Germany

**Correction to: International Journal of Computer Assisted Radiology and Surgery** 10.1007/s11548-023-02892-4

The original version of this article unfortunately contained a mistake. First names and last names of the authors have been swapped.

The correct given name and family name should be

Given Name: Lars

Family Name: Wagner

Given Name: Sven

Family Name: Kolb

Given Name: Christian

Family Name: Looschen

Given Name: Lukas

Family Name: Bernhard

Given Name: Jonas

Family Name: Fuchtmann

Given Name: Maximilian

Family Name: Berlet

Given Name: Johannes

Family Name: Fottner

Given Name: Alois

Family Name: Knoll

Given Name: Dirk

Family Name: Wilhelm

The original article has been corrected.

